# Neuroendocrine tumor in gastric adenoma: a diagnostic pitfall mimicking invasive adenocarcinoma

**DOI:** 10.1186/1746-1596-7-102

**Published:** 2012-08-15

**Authors:** Sun-Mi Lee, Soomin Ahn, Yun Kyung Lee, Kee-Taek Jang, Cheol Keun Park, Kyoung-Mee Kim

**Affiliations:** 1 Department of Pathology, The University of Texas Health Science Center at San Antonio, San Antonio, TX, USA; 2 Department of Pathology, Samsung Medical Center, Sungkyunkwan University School of Medicine, #50, Ilwon-dong, Gangnam-Gu, Seoul, South Korea

**Keywords:** Neuroendocrine tumor, Adenoma, Microcarcinoid, Diagnosis

## Abstract

Neuroendocrine tumor (NET) in adenoma of the gastrointestinal tract is a rare mixed glandular-endocrine neoplasm and has uncommonly been described mostly in the colon. Histologically, this tumor is composed of a predominant proportion of benign adenomatous component and a small portion of well-differentiated NE component. Only three cases of NET in gastric adenoma have been reported in the literature. We present 4 cases of NET in gastric adenoma mimicking invasive adenocarcinoma. The NETs were 0.62 mm to 4.1 mm in size and located at the basal lamina propria, muscularis mucosa and submucosa. Histologically, NETs consisted of nests, cords, tubules, and clusters of cells that predominantly interposed between the foveolar base without disturbing the overall polyp architecture. The lesions were completely removed by endoscopic submucosal dissection in three cases and in one case, subtotal gastrectomy was performed because endoscopic biopsy was invasive adenocarcinoma. The patients’ clinical course was uneventful without an evidence of recurrence or metastasis. The recognition of NET in gastric adenoma will help avoid potential diagnostic pitfalls masquerading as invasvie adenocarcinomas posed by their infiltrative pattern into submucosa.

**Virtual slides:**

The virtual slide(s) for this article can be found here: http://www.diagnosticpathology.diagnomx.eu/vs/1688552293761001

## Background

Although localized endocrine cell differentiation in benign or malignant glandular neoplasms of the gastrointestinal tract is relatively common, truely mixed glandular-endocrine neoplasms are rare. These tumors are composed of both glandular component like adenomas and adenocarcinomas and recognizable neuroendocrine tumor compoments. Most mixed glandular-endocrine neoplasms of the stomach are malignant tumors arising in the background of atrophic gastritis [1-5]. The histologic spectrum ranges from amphicrine carcinoma to admixture of adenoma or adenocarcinoma with neroendocrine tumor (NET) or neuroendocrine carcinoma. Only three cases of mixed benign adenoma-NET have been described in the stomach to date [6-8].

Herein, we describe the clinicopathologic features of four NETs in association with gastric adenomas, to further delineate their histologic features and discuss the diagnostic pitfalls. Recognition of this rare tumor is important not only it can be missed easily due to the minute size of the lesion but also it mimicks an invasive adenocarcinoma arising from adenoma, which may cause unnecessary surgery.

## Case presentation

### Clinical summary

***Case 1***. A 64 year-old man was referred to our hospital with a 3.8 cm sized polyp in the high body after a screening upper endoscopy at outside hospital. An elevated polyp was removed in one-piece by endoscopic submucosal dissection. The patient received a repeat endoscopy at one and two year and no remnant lesion was noted.

***Case 2.*** A 63 year-old man presented with indigestion and abdominal discomfort. Upper gastrointestinal endoscopy revealed a 0.5 cm sized elevated nodule in the lesser curvature of the antrum. The lesion was biopsied initially and endoscopioc submucosal dissection was done. The patient repeated upper gastrointestinal endoscopy at one and two year and no residual lesion was seen.

***Case 3***. A 52 year-old man was found out to have a 1.5 cm sized polyp in the lower body of the stomach during staging work up for rectal cancer. The patient underwent endoscopic submucosal dissection and the lesion was completely removed. The resected segment of rectum revealed a moderately differentiated adenocarcinoma and a incidentally found, separately located 0.6 cm sized NET. He received a repeat upper and lower gastrointestinal endoscopy at two year without any evidence of recurrence.

***Case 4***. A 65 year-old man with no family hstory of multiple endocrine neoplasia presented with chronic dyspepsia. Upper gastrointestinal endoscopy and computed tomography scan revealed a 5 cm sized gastric mass in the lesser curvature aspect of the body. Initial endoscopic biopsy diagnosis at the local clinic was well differentiated adenocarcinoma. He underwent a subtotal gastrectomy and was found to have a 5.5 cm tubulovillous adenoma. Unexpectedly, small nests of tumor cells forming a mass infiltrated into the submucosa with an invasion depth of 800 μm. All sixty regional lymph nodes procured from the resected specimen were free of tumor. The patient has no evidence of recurrence or metastasis during 12 years of follow-up.

### Histopathology

**Figure 1:**
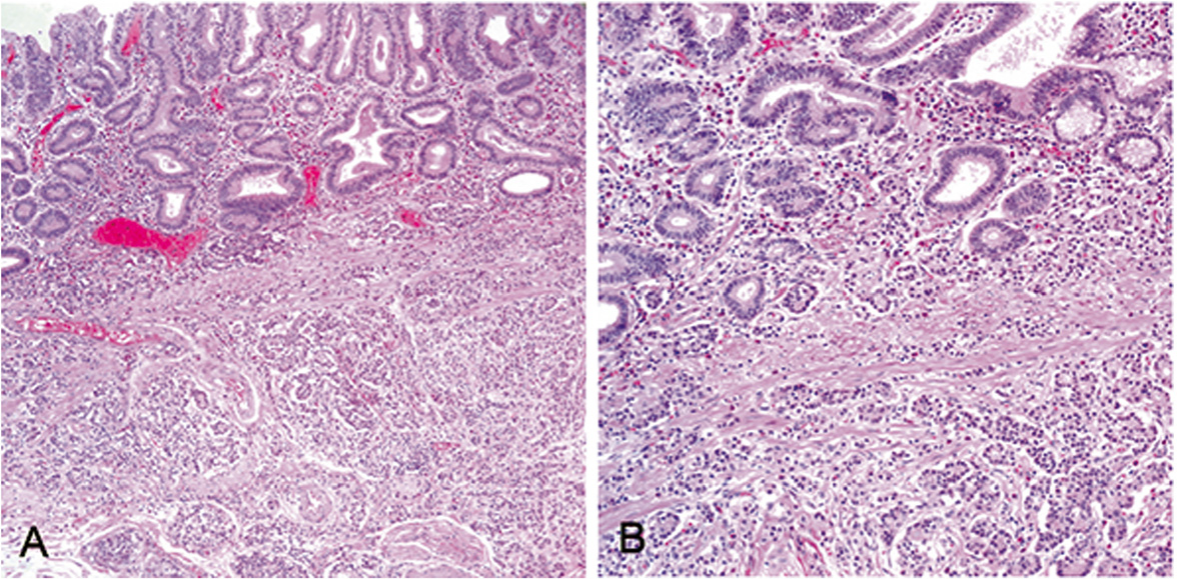
**Neuroendocrine tumor with an organoid growth pattern infiltrating into the submucosa within a tubular adenoma (A).** Higher magnification showing small tumor cells in the deeper part of mucosa and submucosa (**B**).

**Figure 2:**
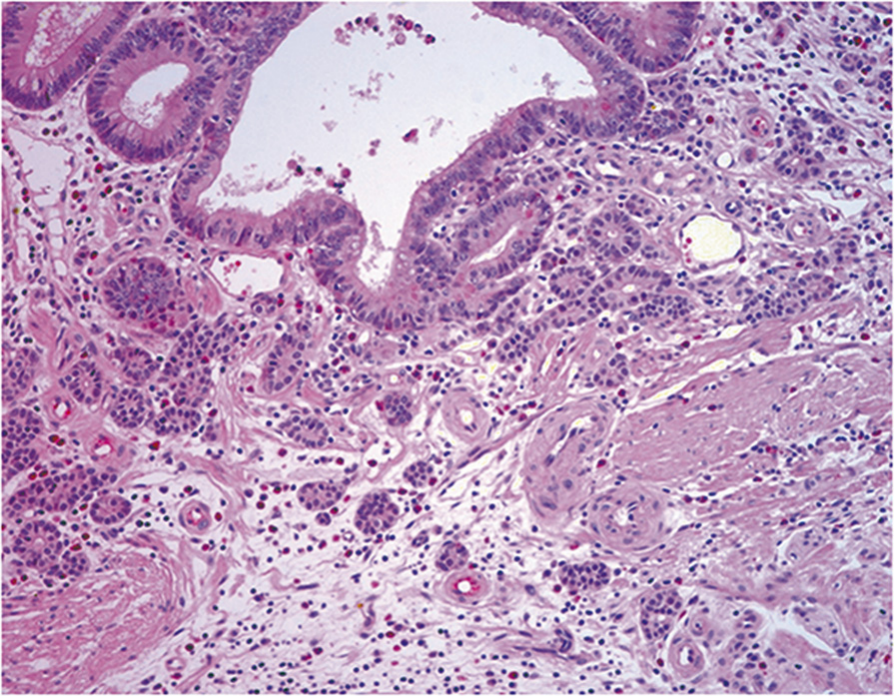
In the base of polyps, neuroendocrine tumor cells appeared to bud off from the basal epithelium of adenomatous glands into the lamina propria with an angulated glandular appearance.

**Figure 3:**
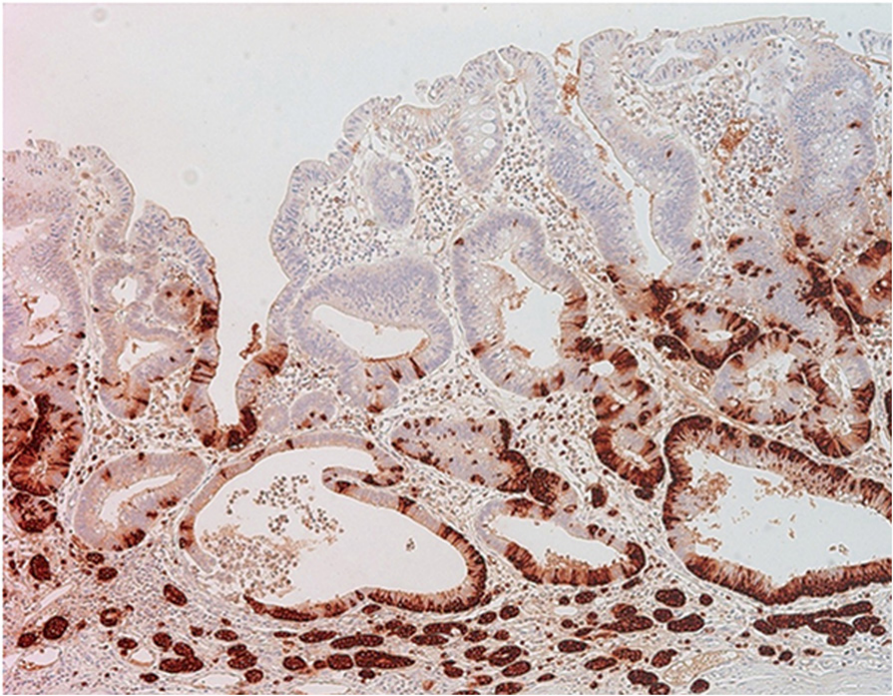
Immunohistochemistry for chromogranin showing positive in glands in the lower part of adenoma and neuroendocrine tumor cells.

The histology of all four cases was similar. The clinicopathologic details of four cases are listed in Table 1. The glandular components of four cases were tubular adenomas with low- and high-grade dysplasia which consisted of tubular-shaped glands lined by psuedostratified columnar epithelium with elongated hyperchromatic nuclei having coarse chromatin and occasional mitotic figures. In all cases, neuroendocrine components represented only a small portion of the adenoma, which were defined as NETs. The NETs were located in the lamina propria and muscularis mucosa in three cases. In one case, the NET cells infiltrated into the submucosa (Figure 1). All the NET component of each case consisted of solid nests, clusters, tubules and cords of cells that predominantly interposed between the foveolar base without disturbing the overall polyp architecture. The cells had abundant eosinophilic granular cytoplasm and cytologically bland, central and round nuclei with finely stippled chromatin. Nucleoli were absent or inconspicous and mitotic figures or necrosis were not observed. Both adenoma and NET components intermingled and merged together, and in some areas, both components were difficult to distiniguish from one another. In the base of polyps, the NET component appeared to bud off from the basal epithelium of adenoma into the lamina propria (Figure 2). The transitional zones demonstrated individual polygonal cells and tiny nests of angulated cells which had enlarged nuclei with occasional prominent nucleoli and scanty mitotic activity. Multiple foci of endocrine cell proliferation with linear growth pattern were also seen adjacent to the NETs within the adenomas. The surrounding non-neoplastic gastric mucosa demonstrated diffuse atrophic gastritis with intestinal metaplasia. Immunohistochemical studies showed a biphasic staining pattern; the adenomas were negative for neuroendocrine markers including synaptophysin, chromogranin and CD56, while the neuroendocrine components were positive. However, in the basal part of adenomas, neuroendocrine markers were focally positive (Figure 3).

**Table 1:** Clinical and Pathologic Characteristics of cases with gastric adenoma-neuroendocrine tumor

**Case**	**Age (years)**	**Sex**	**Location**	**Procedure**	**Size (mm)**	**Depth of invasion**	**Pathologic**	**Follow-up Time**
							**Diagnosis**	
1	64	M	Body, PW	ESD	0.62	MM	TA and NET	2 years
2	63	M	Body, PW	ESD	4.0	LP	TA and NET	2 years
3	52	M	Body, PW	ESD	1.8	MM	TA and NET	2 years
4	65	M	Body, LC	Subtotal gastrectomy	4.1	SM	TVA with focal AC and NET	12 years

*M* indicates male; *PW*, posterior wall; *LC*, lesser curvature; *ESD*, endoscopic submucosal dissection; *MM*, muscularis mucosae; *LP*, lamina propria; *SM,* submucosa; *TA*, tubular adenoma; *TVA*, tubulovillous adenoma; *AC,* adenocarcinoma; *NET*, neuroendocrine tumor.

## Discussion

The concept of a mixed adenoma-NET tumor of the gastrointestinal tract was first introduced by Moyana and Murphy in 1988 [9]. Mixed glandular-endocrine neoplasms in the gastrointestinal tract have been categorized into three subtypes depending on their predominant proportions of each component by Levin et al.: composite tumors, collision tumors and amphicrine tumors [10]. Recently, Pulitzer M et al. described “microcarcinoids”, minute NETs, in benign adenomas in cases that the neuroendocrine component is not enough to qualify for at least one third of the tumor volume, occupying a minute region of the adenomatous polyp like ours [11]. The size of NETs in their study ranged from 5 mm to 20 mm.

We report four rare cases of gastric adenomas containing NETs with an infiltrative growth pattern mimicking adenocarcinoma. The most important reason to recognize NET in a gastric adenoma is to avoid misdiagnosis of this rare lesion as an adenocarcinoma arising from adenoma, which is more common. In our cases, some areas at the base of a polyp showed an intermixture and mergence of adenomatous glands and neuroendocrine cells, which can be misled as tumor cell pleomophism found in adenocarcinoma. These lesions appeared to be arisen from the basal epithelium of adenomas showing “budding-off” angulated glands and infiltrated into the muscularis mucosa or submucosa.

After confirmation of NET in our cases by immunohistochemistry, there was an issue to classify the proliferation of the neuroendocrine cells within adenomas depending on its size and distribution. Until now, no definite consensus on the histopathologic classification of proliferative endocrine cell lesion has been established. Solcia et al. has proposed the subclassification based on size, growth pattern, and numbers of endocrine cells within glands or crypt as follows: simple hyperplasia; linear hyperplasia; micronodular hyperplasia; dysplasia (< 0.5 mm in diameter); NET (> 0.5 mm) [12]. Neuroendocrine components in our cases ranged from 0.62 mm to 4.1 mm. Based on Solcia et al’s classification, we could define our cases as NET.

The prognosis of benign mixed adenoma-NET of the stomach can not be completley determined due to the rarity of cases. Complete removal of an adenoma would be considered curative whereas the combined NET component would be a main predictive factor to determine the patients’ prognosis. In our four cases, three cases showed NETs limited in the lamina propria and focally extended into the muscularis mucosa without any evidence of local recurrence or metastasis. A large series by Soga et al. demonstrated that small submucosal NETs of the stomach had a relatively high metastatic rates [13]. This study showed that the metastasis in the early-stage NETs of the stomach is correlated with not only the submucosal invasion but also the size, which is more than 10 mm. One of our 4 cases was 4.1 mm in greatest dimension and showed submucosal invasion. However, this patient underwent a subtotal gastrectomy and his sixty regional lymph nodes were free of tumor and no metastasis has been identified in a long term close follow-up for 12 years.

## Conclusion

In summary, NET may be found in adenomas of the stomach incidentally, has characteristic morphologic features and immunoexpressions as neuroendocrine cells, and can be under or overdiagnosed due to the small size of the the lesion and overlapping histology with adenocarcinoma. This lesion can be completely treated by polypectomy or endoscopic resection technique without recurrence or metastasis. However, if the NET involves the submucosal layer, close follow-up is recommended for a relatively higher metastatic rate.

We described four rare cases of NET arising from gastric adenoma and followed benign clinical courses. Recognition of this rare entity is important for accurate diagnosis and treatment.

## Consent

Written informed consents were obtained from the patients for publication of this case report and any accompanying images.

## Competing interests

The authors have no potential conflicts of interest to disclose.

## Authors’ contributions

SMA, YKL, KTJ, and CKP made contributions to acquisition of clinical data, and analysis of the histologic features by H&E stain and immunohistochemistry. SML drafted the manuscript. KMK revised manuscript critically for important intellectual content and had given final approval of the version to be published. All authors read and approved the final manuscript.
